# Impedimetric Detection of Femtomolar Levels of Interleukin 6, Interleukin 8, and Tumor Necrosis Factor Alpha Based on Thermally Modified Nanotubular Titanium Dioxide Arrays

**DOI:** 10.3390/nano10122399

**Published:** 2020-11-30

**Authors:** Katarzyna Arkusz, Ewa Paradowska

**Affiliations:** Department of Biomedical Engineering, Faculty of Mechanical Engineering, University of Zielona Gora, Licealna 9 Street, 65-417 Zielona Gora, Poland; e.paradowska@ibem.uz.zgora.pl

**Keywords:** titanium dioxide nanotubes, interleukin 6, interleukin 8, tumor necrosis factor, immunosensor, electrochemical impedance spectroscopy

## Abstract

An inexpensive, easy to prepare, and label-free electrochemical impedance spectroscopy-based biosensor has been developed for the selective detection of human interleukin 6 (IL-6), interleukin 8 (CXCL8, IL-8), and tumor necrosis factor (TNFα)—potential inflammatory cancer biomarkers. We describe a, so far, newly developed and unexplored method to immobilize antibodies onto a titanium dioxide nanotube (TNT) array by physical adsorption. Immobilization of anti-IL-6, anti-IL-8, and anti-TNFα on TNT and the detection of human IL-6, IL-8, and TNFα were examined using electrochemical impedance spectroscopy (EIS). The impedimetric immunosensor demonstrates good selectivity and high sensitivity against human biomarker analytes and can detect IL-6, IL-8, and TNFα at concentrations as low as 5 pg/mL, equivalent to the standard concentration of these proteins in human blood. The calibration curves evidenced that elaborated biosensors are sensitive to three cytokines within 5 ÷ 2500 pg/mL in the 0.01 M phosphate-buffered saline solution (pH 7.4).

## 1. Introduction

Chronic inflammatory reactions have received much attention in recent years due to wrong effector mechanisms or immune response [1]. Both may be harmful or cause life-threatening diseases such as cancer, septic shock, aortic valve stenosis, Alzheimer’s, heart attack, kidney failure, and surgical complication. Proinflammatory cytokines first appear in the area of damaged tissue (i.e., cancer cell division) and play an important role in transmitting signals into leukocytes through endothelium activation [2]. Several publications [3,4,5] have appeared in recent years documenting the increased expression of interleukin 8 (CXCL8, IL-8), interleukin 6 (IL-6), tumor necrosis factor (TNFα) in cancer cells, infiltrating neutrophils, endothelial cells and tumor-associated macrophages.

IL-8 affects gene expression (via regulation of numerous transcription factor activities), modulates the cellular proteome, and affects cell cytoskeleton organization. IL-8 signaling increases the proliferation and survival of endothelial cells, promotes angiogenic responses, and potentiates endothelial cell migration and the infiltration of neutrophils in the affected area [5]. This confirms the significant role of IL-8 within the inflammatory microenvironment. Accordingly, IL-8 expression correlates with the course of septic shock, angiogenesis, and metastasis of tumors.

Several works [4] recently showed the increased expression of IL-6 and/or its receptors in bone metabolism, hematopoiesis, pathogenesis, inflammatory responses, and cancer progression. The increased concentration of IL-6 is observed in lung, uterus, kidney, colon, breast, and ovarian cancers. It was confirmed that its level is correlated with the size and staging of the tumor [6].

The biological function of TNFα is extensive due to membrane receptors affecting various cells. The biggest influence was observed in the immunological reactions and tumor cells [4]. Many authors demonstrated the anticancer effects of TNFα due to tumor cell proliferation inhibition, stimulating apoptosis, and increased cell differentiation. TNFα inhibits angiogenesis and accelerates the clotting of blood feeding the tumor. However, not all types of cancers occur with the above-described phenomena. Hairy cell leukemia, or chronic lymphocytic leukemia B-ventricular, exhibits the severity of the disease due to the increased TNFα concentration [7].

Due to the extensive biological significance of IL-6, IL-8, and TNFα, fast and common detection/monitoring of these cytokines in the human body are needed.

Current methods for detection of IL-6, IL-8, and TNFα are based on enzyme-linked immunosorbent assay (ELISA) tests [8,9]. These methods require sophisticated instrumentation, aseptic handling, highly qualified personnel. In addition, these techniques are time-consuming and costly. For this reason, research on new biosensors has become very popular in recent years. Previous research [10,11,12,13,14,15,16,17,18,19,20,21,22,23,24,25,26,27] has been carried out to obtain high sensitivity and specificity for direct detection of selected antigens using various commonly available methods, such as electrochemical, optical, fluorometric, radioimmunoassay. Recently elaborated IL-6, IL-8, and TNFα biosensors [10,11,12,13,14,15,16,17,18,19,20,21,22,23,24,25,26,27] are based on gold, combination tapered fiber, polysilicon nanowire, dendrimers, and other nanomaterials. The lowest detection limits of IL-6, IL-8 and TNFα were reached at 0.01 [21], 32 [23], and 0.48 pg/mL [25], respectively. These detection limits were achieved using confocal optics [21], fluorescence microscopy [23], and microresonators [18]—costly, and time-consuming methods requiring sample preparation or multiple functionalized biosensor platforms consisting of carbon nanotubes, gold nanoparticles, and SiO_2_ array.

However, recent research, allowing for obtaining a low limit of detection, required a complicated methodology for preparing the electrode. Electrochemical biosensors have recently attracted much attention due to their simple design and high sensitivity [28]. Among all the electrode materials used for biosensing applications, titanium dioxide (TiO_2_) represents an excellent material with good electrical properties, biocompatibility, and excellent bioactivity [29,30,31,32]. The surface topography, and electrical and adsorption properties of titanium nanotubes are important factors for the successful immobilization of biological substances [33,34,35,36]. Due to TNT’s low corrosion potential, the direct immobilization of protein is very simple and effective. Semiconductive titania nanotubes are shown to be excellent substrates for inorganic and biological electrocatalysts. TNT formed on Ti, or its alloy is ready to use as a biosensor electrode and has good implantology properties [37]. 

Some already published examples of using Ti/TiO_2_ layers as the biosensor platforms are very promising [38,39,40,41,42,43]. The first attempt at hydrogen peroxide (H_2_O_2_) biosensor was made by Liu [38]. The detection limit of the elaborated biosensor was 1.2 µM using horseradish peroxidase with thionine and amperometric detection. Further studies on the detection of H_2_O_2_ using the TNT array were carried out by Kafi [39], Guo [40], Chen [41], Kafi [42], and increased the LOD: 2, 0.92, 0.18, and 0.08 µM, respectively. TiO_2_ nanotubes have also been investigated as an electrode material for glucose detection [43]. The lowest detection limit of glucose was reached at 1.5 µM [43] using glucose oxidase, immobilized via glutaraldehyde on polypyrrole-modified TNT by amperometric technique.

Ever since the development of the first biosensor based on TNT, much attention has been focused on improving the response performances of enzyme electrodes for biosensor applications. The authors used mediators like chitosan [44], hemoglobin [45], and methylene blue [46], and functionalized TNT by gold [34,35], silver [33], glassy carbon [47], or used processes of visible-light-activation [48], and carbonization [49]. The TNT based biosensor was also used for the detection of methanol electrooxidation [50], luminol [51], penicillin-binding protein [52], and atrazine [53].

In the present study, we have developed sensitivity, simple, label-free, and easy to use in point-of-care immunosensor for IL-6, IL-8, and TNFα detection, based on the titanium dioxide nanotube-based nanoelectrode. Thermal modification, electrochemical, and adsorption analysis explain the physisorption mechanism of the antibody on the TNT layer and substantially supplement the existing literature reports. A newly elaborated method to immobilize antibodies onto TNT by physical adsorption has allowed us to detect IL-6, IL-8, and TNFα protein at 5 pg/mL (192, 625, and 295 fM, respectively) in phosphate buffered saline (PBS). The obtained limit of detection is lower than in the standard ELISA kit, matching the physiological level, whereas the higher concentration of cytokines includes the levels corresponding to inflammation states. The total time of determining the concentration of IL-6, IL-8, and TNFα was reduced from 6 to 2 h.

## 2. Materials and Methods

### 2.1. Materials

Titanium foil (thickness 0.5 mm, 99.99% trace metal basis), phosphate buffered saline (PBS, 0.01 M, pH 7.4), ammonium chloride (NH_4_F, 99.5%), and ethylene glycol (C_2_H_6_O_2,_ 99.8%), were purchased from Sigma-Aldrich (St. Louis, MO, USA). The ELISA kits for human IL-6 (ELH-IL6-001), IL-8 (ab100575) and TNFα (ET2010-1) protein were purchased from RayBiotech (Peachtree Corners, GA, USA), Abcam (Cambridge, UK), AssayPro (St. Charles, MO, USA), respectively, and stored at 4 °C to prevent protein denaturation. The impedimetric biosensor detection scheme uses monoclonal antibodies (Mab) that form a pair of sandwich assays in human cytokines.

All electrochemical measurements—i.e., anodization of titanium foil, open circuit potential (OCP), and electrochemical impedance spectroscopy (EIS) measurements—were carried out with the AutoLab PGSTAT 302N potentiostat/galvanostat (Eco Chemie, Utrecht, The Netherlands) equipped with a voltage multiplier (30 V) using the standard three-electrode configuration with a titanium electrode and Ti/TNT as the working electrode, the standard Ag/AgCl^−^ silver chloride electrode (E_Ag/AgCl_ = 0.222 V) as the reference electrode, and a platinum foil as the auxiliary electrode at 25 ± 1 °C.

### 2.2. TiO_2_ Nanotubes Fabrication

The titanium foil was sonicated in acetone, ethanol, and distilled water and dried in nitrogen. The formation of nanotube oxide layers was performed by electrochemical anodizing in various ethylene glycol solution concentrations with 0.65% wt. NH_4_F. The formation process consists of two stages: the first potentiodynamic and the second potentiostatic. In the beginning, the foil was polarized up to 17 V with a scan rate of 0.5 V/s and then was kept at that potential in the same electrolyte for a further 3750 s. Field emission scanning electron microscopy (FESEM, JEOL JSM-7600F, Tokyo, Japan) and energy-dispersive X-ray spectroscopy (EDS, INCA, Oxford Instruments, Oxford, UK) was used to investigate surface morphology and chemical composition.

### 2.3. Thermal Modification of TNT

The obtained TNT layers were annealed in argon at 550 °C for 2 h with the heating rate of 6 °C/min to the desired annealing temperature. Field emission scanning electron microscopy (FESEM, JEOL JSM-7600F, Tokyo, Japan) and energy-dispersive X-ray spectroscopy (EDS, INCA, Oxford Instruments, Oxford, UK) were used to investigate surface morphology and chemical composition.

### 2.4. Evaluation of the Open Circuit Potential (OCP) and Contact Angle of TNT

The OPC measurements were performed using Ti/TNT samples before and after thermal modification immersed in PBS solution (0.01 M, 50 mL, pH 7.4) for 1800 s.

Dynamic contact angle measurements were performed using distilled water at room temperature using a PG-3 Goniometer equipped with a microscope, a camera, and an integrated micro-pump, which deliver accurate droplets in 0.5 µL steps.

### 2.5. Functionalization of TNT Layers with Antibodies and IL-6, IL-8, TNFα Detection

IL-6, IL-8, TNFα were immobilized on the biosensor platform using the simple drop coating method at room temperature. Annealed in argon, Ti/TNT samples were incubated with 10 μL of IL-6, IL-8, TNFα antibody solutions for different times and then treated with 10 μL of IL-6, IL-8, TNFα antigens solutions of various concentrations ranging from 0 to 2500 pg/mL. After the immobilization process, each sample was washed with a buffer solution (3 × 100 µL) to remove non-specifically bound biomolecules. Spectrophotometric measurements were performed using a Hitachi UV-2310II Spectrophotometer at a wavelength of 450 nm.

The response of impedimetric immunosensor was recorded using Electrochemical Impedance Spectroscopy (EIS) method. Impedance analysis was performed over a frequency range from 0.1 Hz to 100 kHz (10 points/decade), at zero DC potential, with an AC amplitude of 10 mV and an integration time of 10 s. The effects of electrolyte pH on the response of elaborated biosensors were studied using IL-6, IL-8, and TNFα antigens with a concentration of 300 pg/mL.

## 3. Results and Discussion

### 3.1. Characteristics of Titanium Dioxide Nanotubes

**Figure 1:**
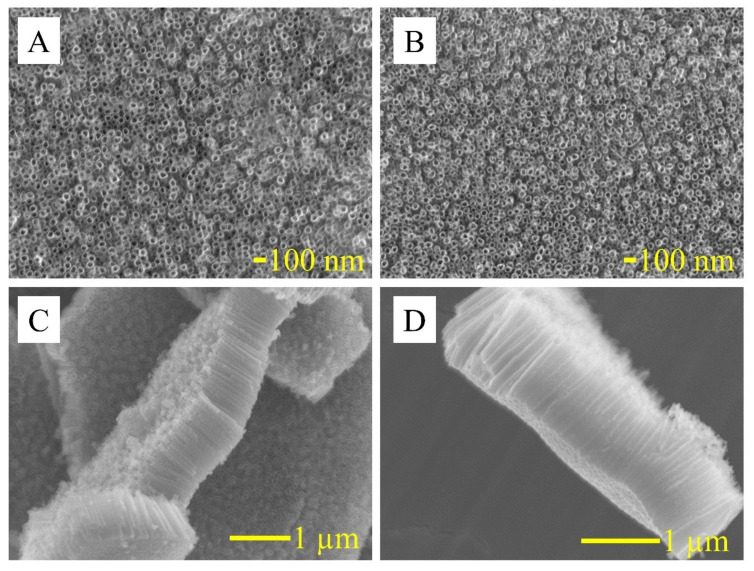
SEM images of top view (**A,B**), and cross view (**C,D**) of TNT (**A,C**), and TNT annealed in argon (**B,D**) at 550 °C during 2 h.

As shown in Figure 1, the TNT formed in the anodizing process, according to the applied parameters, results in a uniform 1000 ± 100 nm thick layer of vertically aligned nanotubes (Figure 1a) with a diameter of about 50 ± 5 nm. SEM pictures show homogenous arrays of opened from the top, closed at the bottom, and vertically oriented nanotubes completely covering the titanium foil. No damage was observed on the TNT layer after annealing (Figure 1b). Obtained SEM images confirm that the thermal modification does not influence TNT layer homogeneity and morphology—its height and diameter were not changed.

The height and diameter of TNT were chosen based on previous electrochemical analysis, indicating the most favorable sensing and biosensing properties [31]. This structure is characterized by OCP values close to zero, one of the smallest impedance module, and a high Ipa/Ipc ratio for potassium ferricyanide, which confirms good conductivity and good adsorption properties of this structure [29]. Moreover, titanium nanotubes annealed at a temperature of 550 °C in an argon atmosphere have more anatase structures that promote biological element adsorption [32]. However, previous experiments do not attempt antibody immobilization on the TNT surface.

The EDS microanalysis results for samples formed in ethylene glycol and thermally modified in argon are presented in Table 1. According to the formation conditions, the results showed the difference in concentrations of elements in the TiO_2_ nanotubes layer. After thermal treatment, fluoride is not identified, and obtained titanium oxide is non-stoichiometric. Fluoride uptake caused an increase in oxygen content in TNT annealed in argon (33.9%). Annealing in argon could change the ingredients of the oxide layer leading to the formation of oxygen vacancies. The possible explanation is reducing titanium ions from Ti^4+^ to Ti^3+^ ions and the formation of oxygen vacancies [32,33,34,35].

**Figure 2:**
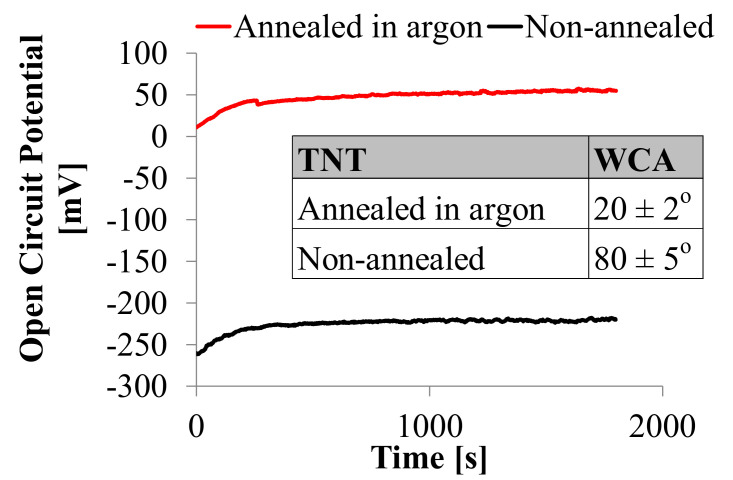
Water contact angle (WCA) and open circuit potential (OCP) measurements of TNT layers (diameter 50 ± 5 nm, 1000 ± 100 nm thickness) before and after annealing in argon in temperature 550 °C for 2 h.

The Open Circuit Potential (OCP) curves (Figure 2) show stable potential recorded values during the 1800 s. Small current oscillations seen in OCP curves for TNT formed in ethylene glycol electrolyte confirm ongoing oxidation and metastable nature of nanotubular oxide layers [29]. Annealing in argon causes the increase in the OCP value (E_OCP_ = 55.1 ± 4.2 mV) for Ti/TNT layers compared to the OCP value for non-annealed samples (E_OCP_ = −220.1 ± 12.1 mV). The contact angle measurements (WCA) carried out on the annealed samples for doubly ionized (DI) water confirmed that all obtained surface layers are hydrophilic. The value of the contact angle presented in Figure 2 for the non-annealed TiO_2_ nanotubes, which amounts to 20° ± 2° after annealing in argon, drops to 80° ± 5°.

Anodic titania oxides exhibit semiconductive properties attributed to defects: oxygen vacancies, Ti^3+^ interstitials, and solvent components and impurities. TNT’s conductive properties may be improved by annealing in controlled atmospheres, which causes the semiconductor material to obtain sufficiently higher electronic and ionic conductivity and improve wettability [33]. More positive values of the open circuit potential are favorable for the protein immobilization on the TNT platform, according to the amino acid residues (e.g., HRP) and human antibodies exhibiting negative charges in PBS (0.01 M, 50 mL, pH 7.4). The negatively charged protein is easily attached to the biosensor’s positive matrix by direct physisorption, resulting from weak electrostatic or van der Waals interaction [54]. The material characterized by WCA values near 90° can affect hydrophobicity and hydrophilicity, which is favorable for the immobilization process. Protein adsorption is stronger on the hydrophobic surface, but hydrophilic material avoids the protein decomposition [55].

The surface topography, electrical, and adsorption properties of titanium nanotubes (TNT) are important factors for the successful immobilization of biological substances. However, chemical modification of a TNT platform using currently available methods (i.e., silanization) often results in the effective immobilization process via covalent binding. Still, this makes the manufacturing of biosensor time-consuming and costly.

Recently, direct immobilization on the TNT surface was confirmed for the chemical compounds that exist as a cation in aqueous solutions [56]. Physisorption by the electrostatic forces of human antibodies onto the TNT surface, taking into account the values of the isoelectric point of human antibodies, requires the surface charge’s positive value. Most biological substances have an isoelectric point lower than 7.4, which results in anions formed in human plasma and serum. Therefore, the immobilization of enzymes and proteins on the surface of TNT requires the presence of mediators [57,58]. Physisorption by the electrostatic forces of human antibodies onto the TNT surface, taking into account the values of the isoelectric point of human antibodies, requires the surface charge’s positive value. The surface treatment was performed before the immobilization process to change TNT’s surface charge from negative to positive.

### 3.2. IL-6, IL-8, TNFα Antibodies Adsorption

**Figure 3:**
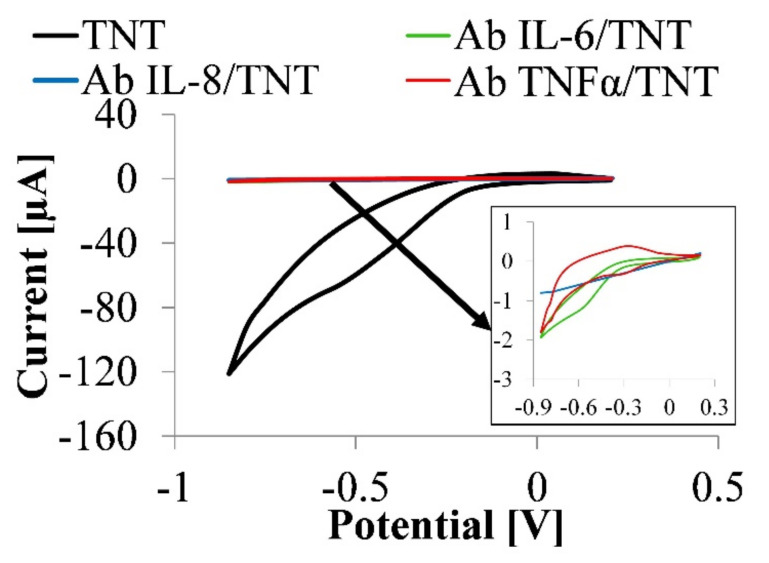
CV curves obtained for the TNT electrode and Ab IL-6, IL-8, TNFα immunoelectrode in 0.01 M PBS solution without any external mediator.

Cyclic voltammetry (CV) studies of TNT and Ab/TNT electrodes in the PBS solution without any external mediator are shown in Figure 3.

It is important to note that the bare TNT platform does not show any redox peak, which is related to the fact that TNT does not have any redox couple for the electron transfer from the reaction site to the electrode. Flat cyclic voltammograms confirm the platform’s stability in the electrolyte and the lack of redox reaction on TNT’s surface. TNT annealed in argon indicates higher current values that prove TNT’s high conductivity and provides a fast electron transfer path.

It is clearly seen that the current response serially decreases (Ab IL-6, IL-8, TNFα) when the electrode is modified with antibodies, as seen in Figure 3. This might be attributed to the insulating Ab protein layers on the electrode, which would hinder the electron transfers. This is related to the fact that the macromolecules of Ab IL-6, IL-8, TNFα restrict the flow of charge carriers. The results indicated that the immunosensing substrate had formed successfully and could be used in the proposed immunoassay.

After understanding the surface charge of TNT, IL-6, IL-8, TNFα antibodies, adsorption was studied. The direct immobilization of IL-6, IL-8, and TNFα antibodies on the TNT surface was performed by the drop technique for 60 min. The shortest time and the immobilization efficiency (% of the antibodies attached to the platform surface) were evaluated based on the spectrophotometric analysis of the slopes on every stage of the immobilization process.

**Figure 4:**
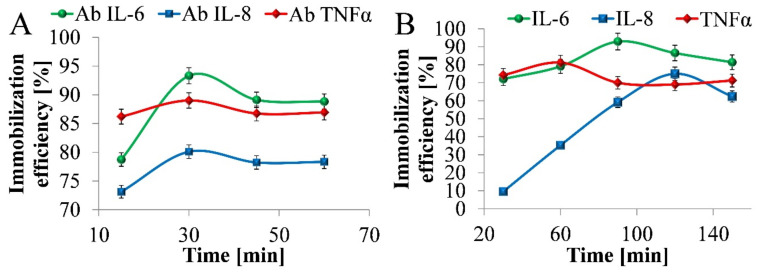
Immobilization efficiency of (**A**) IL-6, IL-8, TNFα antibodies, and (**B**) IL-6, IL-8, TNFα antigen on TNT.

According to ELISA tests, immobilization time was defined as 60 min for each antibody, 120 min for TNFα antigens, and 150 min for IL-6 and IL-8 antigens. The average value of the immobilization efficiency of IL-6, IL-8, TNFα immobilization onto TNT’s surface was 87.5% (Figure 4a), and for IL-6, IL-8, TNFα antigens was 83.1%. This proves the high physical affinity of IL-6, IL-8, TNFα to the prepared platform covered with TNT and confirms TNT’s suitability for effective protein immobilization and biosensing.

For each cytokine, the shortest antibody immobilization time was defined as 30 min and the shortest antigen immobilization time as 90, 120, and 60 min for IL-6, IL-8, and TNFα, respectively. This period was considered sufficient for the effective binding of biomolecules to the TNT matrix based on the performed measurements.

### 3.3. Label-Free Detection of IL-6, IL-8, TNFα using Electrochemical Impedance Spectroscopy

**Figure 5:**
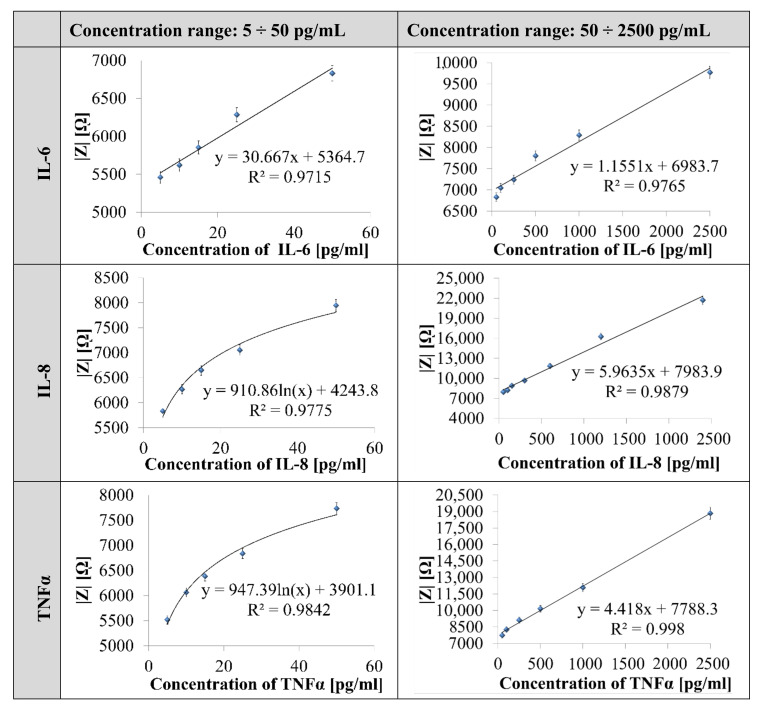
Calibration curve obtained between |Z| and concentration of IL-6, IL-8, and TNFα measured in 0.1 Hz frequency.

**Figure A1:**
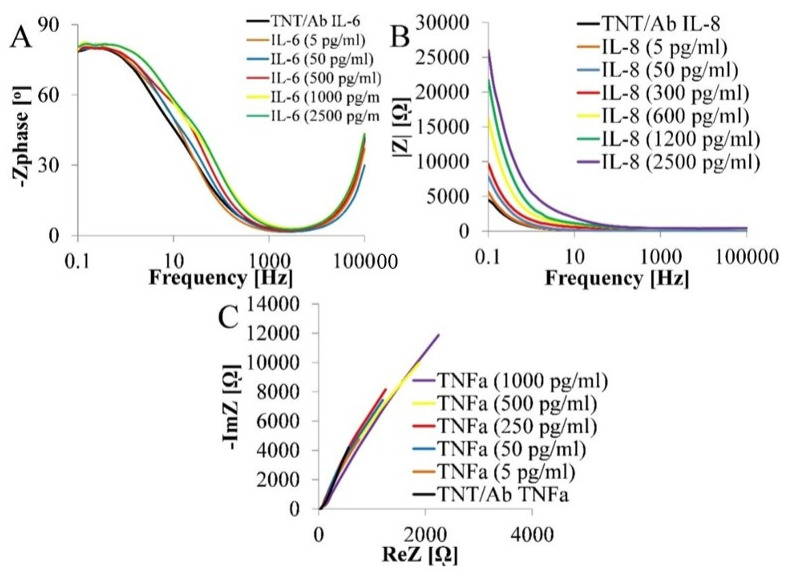
Impedimetric response studies of TNT immunoelectrode incubated with different concentration of IL-6, IL-8, TNFα, presented in Bode representations for IL-6 (**A**) and IL-8 (**B**) and in Nyquist representation for TNFα (**C**).

The impedimetric response of the TNT layer (diameter of 50 ± 5 nm, the height of 1000 ± 100 nm) annealed in argon, immobilized with IL-6, IL-8 and TNFα antibody on the varying concentration of IL-6, IL-8, and TNFα antigens, were analyzed using EIS measurements carried out in the frequency range 10^5^ ÷ 0.10 Hz in PBS (0.01 M, pH 7.4) solution (Appendix A). An example of Nyquist and Bode plots for the determination of IL-6, IL-8, and TNFα are shown in Figure A1 (Appendix A), including the statistical analysis of performed measurements (Table A1), whereas the calibration curves are presented in Figure 5.

Impedance biosensor responses for IL-6, IL-8, and TNFα are presented in the form of calibration curves (Figure 5), created based on the relationship between the parameters of the impedance characteristics of the biosensors and the concentrations of the determined cytokines.

**Figure 6:**
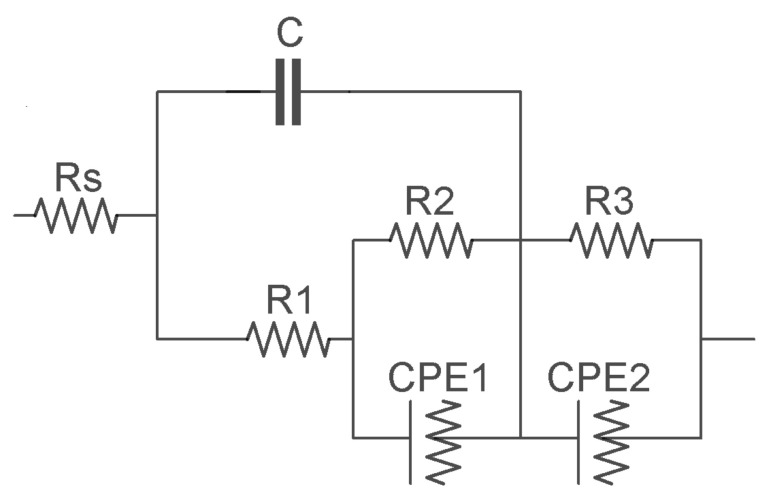
Equivalent circuit for fitting the plots.

The calibration curves (Figure 6) show a linear variation in |Z| from 5458 ± 110 Ω to 9771 ± 195 Ω, from 5831 ± 115 Ω to 21,706 ± 430 Ω, from 5517 ± 110 Ω to 18,834 ± 375 Ω over a wide range of IL-6, IL-8 and TNFα concentration (5–2500 pg/mL), respectively. The straight line is fitted to the data by linear regression analysis defined by the equations with a regression coefficient (R2), shown in each graph. The regression coefficient values are close to 1, indicating good linearity of the calibration curve.

Obtained EIS spectra for TNT/antibody/antigen for the assumed range of concentrations of IL-6, IL-8, TNFα were fitted to the equivalent circuit using Nova 1.8 software. The circuit characterized by the best fit (based on the results of mean square errors ×2 of matching the equivalent circuit using the nonlinear least-squares method) was the one whose graphical form is shown in Figure 6, where Rs—electrolyte resistance; R1—resistance of the porous/nanotubular layer; R2—resistance of the ionic interface between the double layer and the solution; R3—resistance of the biological layer; C1—capacitance of the porous/nanotubular layer; CPE1—double-layer capacitance; CPE2—capacitance of the biological layer.

The obtained results confirm the correlation between the determined cytokine concentration and the parameters of the replacement system (Table 2). As the concentration of human IL-6 increases the electrolyte resistance (Rs) increases, the resistance of the porous layer (R1) increases, and the capacity of the biological layer (CPE2) increases. As the concentration of human IL-8 increases the resistance of the porous layer (R1) increases, the capacity of the porous layer (CPE1) decreases, and surface heterogeneity and roughness (α1) increase. With the increase in the concentration of human TNFα the resistance of the electrolyte (Rs) increases, the resistance of the double layer (R2) increases, and surface heterogeneity and roughness (α1) increase.

### 3.4. Effect of pH of Electrolyte on IL-6, IL-8, TNFα Biosensor Response

**Figure 7:**
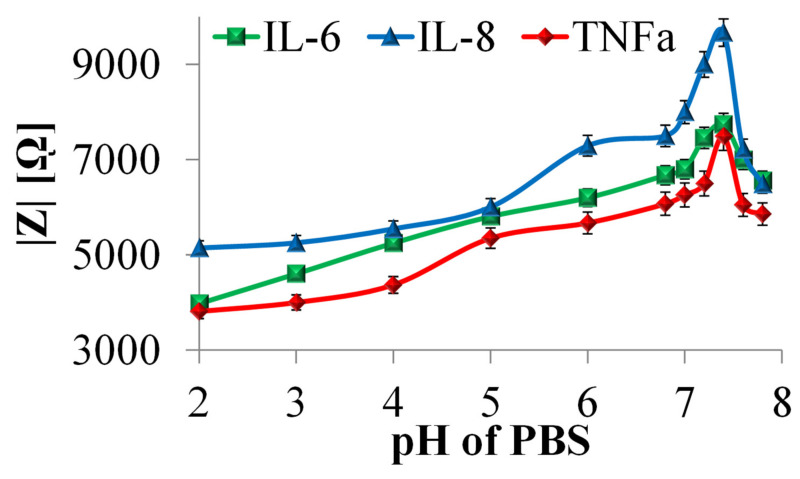
Influence of electrolyte pH on the value of the impedance modulus measured at the frequency of 0.1 Hz of the impedance biosensor IL-6, IL-8 and TNFα, recorded in a 0.01 M PBS solution, using the amplitude of the excitation signal of 10 mV.

The effect of pH on the impedance biosensor response was investigated for the IL-6, IL-8, and TNFα antigens at 300 pg/mL concentrations. Measurements were made in the range from 105 to 0.10 Hz, using the amplitude of the excitation signal of 10 mV, in 5 mL of 0.01 M PBS solution with the following pH values: 2.0; 3.0; 4.0; 5.0; 6.0; 7.0; 7.2 7.4; 7.6; 7.8, at a temperature of 25 °C. The measurements were carried out with at least three-fold repeatability. The curves showing the dependence of the impedance value (biosensor response) on the analyte’s pH are shown in Figure 7.

The curves illustrating the influence of pH electrolyte on the impedimetric biosensor response have a similar course for determining cytokines. In the range of pH 2 ÷ 7.4 for IL-6, IL-8, and TNFα, it was observed that increasing the pH electrolyte results in an increase in the impedance modulus |Z| measured in the frequency of 0.1 Hz. For each of the determined cytokines, the maximum biosensor response was recorded in an electrolyte at pH values of 7.4, which is analogous to human plasma pH. The increase in biosensor response observed in the pH ranges from 6.8 to 7.4 is consistent with literature reports concerning the stability of the antigen–antibody complex and cytokine activity [59,60]. Reverberi and Ward’s research showed that, in the pH range of 6.8–7.4, with increasing pH value, the stability of the antigen–antibody bond increases, and biological substance activity increases. Above the pH value of 7.4, a decrease in the value of the impedance modulus was observed, which may be due to the increase in the number of Na^+^ ions from the PBS alkalizing reagent. This factor has a destabilizing effect on the conformational structure of proteins and prevents antigen–antibody complex formation.

The nature of the dependence of the impedance biosensor response for the detection of IL-6, IL-8, and TNFα concentrations on the pH of the analyte confirms both the sensitivity of the biosensor to a change in analyte pH and the broad spectrum of activity of the analyzed substances and their ability to form antigen–antibody complexes. Considering the potential use of a biosensor to measure cytokine concentration in human plasma, it is advantageous to achieve a maximal biosensor response for an analyte pH corresponding to human plasma.

The detection of selected markers with the use of biosensors, described in the literature (Table 3), is a long-term process due to the multi-stage nature of its preparation [1,2,3,4,5,6,7,8,9,10,11,12,13,14]. The presented biosensors require the use of additional surface functionalization processes, such as silanization [3], surface modification with EDC/NHS [6,7,8,12], Merkapto [8], APTES [4,9], or marking antibodies with biotin/streptavidin [9,13,14]. In addition, the immobilization time of antibodies is equal to night [1,2], 12 [10], 6 [3], 5 [12], 4 [4], and 3 h [6,9], and antigens 6 [3] and 2 h [14]. The titanium dioxide nanotube biosensor described in the manuscript does not require the use of additional surface modification processes, which allows for a faster electrode preparation process. Additionally important is the short time (60 min) immobilization of both antibodies and antigens IL-6, IL-8, and TNFα.

## 4. Conclusions

The development of an efficient electrochemical immunosensor to detect IL-6, IL-8, and TNFα using TNT has been successfully demonstrated. The thermal modification of TNT (diameter: 50 ± 5 nm, height: 1000 ± 100 nm) in argon atmosphere results in a change in the value of open circuit potential from negative to positive, increasing the resistive character and decreasing the nonhomogeneity. The successful immobilization of the antibody of IL-6, IL-8, and TNFα onto the TNT surface via physisorption was carried out for 30 min, which is a much shorter time than currently available analytical methods.

The high sensitivity of the prepared substrates to pH changes was observed in impedimetric tests, and the highest impedance modulus values were measured in the analyte at pH 7.4. The obtained results indicate that the tested cytokine antibodies and antigens are not inactivated in a wide pH range, which ensures the universality of the biosensors being developed.

Calibration curves of impedance biosensors for each of the determined cytokines showed that the detection limits for IL-8, IL-6, TNFα are 192, 625, and 295 fM, respectively, which correspond to physiological norms, where the detection range covers clinically important concentrations from 5 to 2500 pg/mL.

## Figures and Tables

**Table 1:** Elemental investigation of titania nanotubes (50 nm diameter, 1000 nm thickness) before and after thermal modification (annealing in argon, in temperature 550 °C for 2 h).

(wt.)	Non-Annealed	Annealed in Argon
Ti (%)	65.9 ± 4.2	66.1 ± 3.1
O (%)	25.6 ± 2.6	33.9 ± 1.5
F (%)	8.6 ± 3.5	-

**Table 2:** Values of equivalent circuit elements obtained for fitting of the experimental data for various concentrations of IL-6, IL-8, TNFα: R^2^—mean square error; R1—resistance of the porous layer; R2—resistance of the ionic interface between the double layer and the solution; CPE1—double-layer capacitance; N1—*n*-value of a CPE1.

IL-6 (pg/mL)	χ^2^	Rs (ῼ)	R1 (ῼ)	CPE2 (Mho)
0	0.025	33.5	427.4	7.54 × 10^−9^
5	0.077	39.8	319.7	5.22 × 10^−9^
10	0.022	54.2	49.9	4.46 × 10^−9^
25	0.049	57.8	13.9	2.37 × 10^−6^
50	0.016	68.1	2.5	3.43 × 10^−4^
100	0.043	80.6	11.5	2.35 × 10^−4^
250	0.092	100.9	189.2	2.25 × 10^−4^
500	0.094	110.6	1.10 × 10^12^	1.80 × 10^−3^
1000	0.050	117.1	1.10 × 10^12^	0.67
2500	0.040	140.4	1.10 × 10^12^	4.71
**IL-8 (pg/mL)**	**χ^2^**	**R1 (ῼ)**	**CPE1 (Mho) × 10^−4^**	**α1**
0	0.025	54.8	3.1	0.439
5	0.020	83.6	2.8	0.772
10	0.026	88.4	2.7	0.783
25	0.097	110.9	1.7	0.797
50	0.044	113.8	1.3	0.820
100	0.026	118.4	1.2	0.827
150	0.087	308.1	0.14	0.834
300	0.093	332.7	0.15	0.838
600	0.041	474.9	0.0086	0.863
1200	0.016	631.1	0.0088	0.885
2500	0.046	27,553	0.00026	0.974
**TNFα (pg/mL)**	**χ^2^**	**Rs (ῼ)**	**R2 (ῼ)**	**α1**
0	0.056	28.1	3.18	0.765
5	0.019	55.9	70.4	0.780
10	0.094	73.6	72.8	0.792
25	0.053	91.9	91.3	0.837
50	0.026	101.9	103.1	0.860
100	0.035	108.6	152.0	0.863
250	0.015	123.4	181.4	0.871
500	0.064	140.5	247.4	0.899
1000	0.014	196.4	8511.0	0.980
2500	0.047	272.4	17,332.0	0.987

**Table 3:** Properties of different types of biosensor for detection of IL-6, IL-8, and TNFα.

Target	Composition of Sensitive Film	Detection Method	Limit of Detection	Linear Range	Ref.
IL-6	Au rods/Ab^1^	Potentiometric	10 ng/mL	-	[10]
	APCPG/CC/Ab/Ag/Ab1-Strepravidin-alkaline	Amperometric	0.41 pg/mL	0 ÷ 400 pg/mL	[11]
	ZnO/SiO_2_/Si-Ab	SAW	-	20 ng/mL÷ 2 µg/mL	[12]
	Combination tapered fiber-optic biosensor (CTFOB)/Ab	Optic	0.12 ng/mL	40 pg/mL÷100 ng/mL	[13]
	SWCT-Ab1/Ag/Ab2-HRP	Amperometric	0.05 pg/mL	0.5÷5 pg/mL	[14]
	ITO/AuNP/PDOP Poly-dopamine -Ab-Ag-Ab1-HRP/Ab2/CNT	Amperometric	1 pg/mL	4÷800 pg/mL	[15]
	SWCNTforests-Ab1/Ag/Ab2-Ru(bpy)_3_^2+^-silica	ECL	0.25 pg/mL	-	[16]
	Au-Ab1/Ag/Ab2-Poly-HRP	Amperometric	10 fg/mL	10÷1300 fg/mL	[17]
	Magnetic-photocatalytic hybrid nanoparticles with a highly crystalline TiO_2_ shell-Ab	Microresonators	0.1 pg/mL	-	[18]
	SiO_2_/Si/ CNT/AuNP-Ab	Impedimetric	0.01 fg/mL	0.01÷100 fg /ml	[19]
	SWCNT-Ab	FET	1.37 pg/mL	-	[20]
	CNT/AuNP-Ab	Impedimetric	0.01 fg/mL	0.00001÷0.1 pg/mL	[21]
IL-8	PEG/Au/non-antibody capture proteinbased on a cystatin scaffold	Impedimetric	90 fg/mL	900 fg/mL÷900 ng/mL	[22]
	ELISA surface with capture probe	Optical	32 fg/mL	5 fg/mL÷1 pg/mL	[23]
	Polysilicon nanowire-Ab	-	80 fg/mL	-	[24]
TNFα	Dendrimer-based PEG/Ab/Ag/Ab2-HRP	Optical	0.48 pg/mL	0 ÷ 300 pg/mL	[25]
	MB labeled TNFα aptamer	Optical	10 ng/mL	10 ÷ 100 ng/mL	[26]
	Au-Si-dithiobis-succinimidyl propionate	Impedimetric	1 pg/mL	1 pg/mL÷1 ng/mL	[27]

^1^Ab – antibody, Ag – antigen, APCPG – 3-aminopropyl-modified controlled-pore glass, CC – central channel, MB – Methylene Blue, HRP –Horseradish Peroxidase, Au- gold, AuNP – gold nanoparticles, SWCT - single-well chemical tracer, CNT – carbon nanotubes, PEG – Poly(ethylene glycol), SAW – Surface acoustic wave, ECL – Electrochemiluminescence method, FET - Field-Effect Transistor.

## Appendix A

The impedimetric response of TNT layer (diameter of 50 ± 5 nm, the height of 1000 ± 100 nm) annealed in argon, immobilized with IL-6, IL-8 and TNFα antibody on the varying concentration of IL-6, IL-8, and TNFα antigens were analyzed using EIS measurements carried out in frequency range 10^5^÷0.10 Hz in PBS (0.01 M, pH 7,4) solution. An example of Nyquist and Bode plots for the determination of IL-6, IL-8, and TNFα were shown in Figure A1. Each measurement was performed with a minimum of 3-times repeatability. To calculated the values of standard deviation (SD) relative standard deviation (RSD) for electrochemical parameter (EIS) the equations were used accordingly (1), (2) where Χi – each of the values of the data for 3 samples, Χ – the mean of Χi, n – the number of data points equal to 3. The results of statistical analysis were shown in Table A1.

(1) $$SD=\sqrt{\frac{\sum_{i=1}^{n}{\left(Χi-Χ\right)}^{2}}{n-1}}$$

(2) $$\mathrm{RSD}=\frac{SD}{Χ}$$

The phase shift angle values recorded at the lowest frequency (0.1 Hz) presented in Bode’s graphs (Figure A1) range from 50° to 90° for each of the determined cytokines (Table A1). Correlations between its values (measured at a frequency of 0.1 Hz) and the determined IL-8 antigen concentrations were observed (Figure A1B). With the increase in the concentration of the determined cytokine, the value of the phase angle decreases. Along with the increase in the concentrations of antigens: IL-8 and TNFα, the phase angle values for the frequency of 0.1 Hz decrease, which proves that the heterogeneity of the TNT surface decreases due to combining the antigen with antibodies immobilized on the surface of TNT. Forming antigen-antibody complexes, filling the pores, reduce its porosity.

Data illustrating changes in the phase angle (Table A1) for high concentrations of the determined cytokines IL-6, IL-8 and TNFα (concentrations greater than 250 pg/mL for Il-6, 600 pg/mL for IL-8, and 1000 pg/mL for TNFα) indicate the existence of two-time constants, which proves the presence of two layers on the electrode surface: the first is the layer of immobilized antibody, the second is the layer of antibody-antigen complexes. Greater heterogeneity (porosity) the surface layer for lower concentrations may result from incomplete antibody layer coverage with antigens. As antigens increase and more antibody-antigen complexes form, it decreases the value of the phase angle determines the layer’s non-uniformity, and its value measured at high frequency (10^5^ Hz) is also lowered, corresponding to the resistance of the electrolyte penetrating the porous electrode surface. The served increasing values of the phase angle for the time constant in the frequency range from 10 to 50 Hz well illustrate its dependence on the increasing concentration of the antigen. It is also confirmed by the correlation between the phase angle values and different concentrations of IL-6 in the studied range.

The curves showing the dependence of the impedance modulus on frequency for different cytokines concentrations: IL-6, IL-8 (Figure 1B), and TNFα are similar. The impedance modulus values recorded at the lowest frequency of 0.1 Hz are in the range from 3000 to 25000 Ω for the determined cytokines (Table A1). The lowest value of the impedance modulus (~3 × 10^3^ Ω) was recorded for the TNT layer with a diameter of 50 nm, height 1000 nm, annealed in argon, and not functionalized. For each of the determined cytokines, an increase in the impedance modulus’s value was observed with the increase in their concentration. This means that as the TNT layer is coated with antibodies and then with increasing antigens concentrations, which results in the formation of antigen-antibody complexes, the impedance modulus of the test layer increases. This tendency corresponds to the characteristics of biological systems covering the electrodes [58].

Nyquist curves of TNT with IL-6, IL-8, and TNFα (Figure A1C) antibodies immobilized on its surface, similarly to before immobilization, have the shape of large semi-circles - characteristic of thin oxide layers. With the increase in the concentration of IL-6, IL-8, TNFα, the angle of the curves and the radius of the circles decreased, which indicates a decrease in the charge exchange resistance in the presence of an increasing number of antigen-antibody bonds. Their presence on the biosensor electrode’s surface may stimulate the exchange of electrons, causing an increase in the electrical conductivity of the layer, thus reducing its resistance.

**Table A1:** Results of electrochemical impedance spectroscopy obtained for the varying concentration of IL-6, IL-8, TNFα immunoelectrode measured in 0.01 M PBS solution, where: SD – standard deviation, RSD—relative standard deviation.

Marker conc. (pg/mL)	|Z| (Ω)	SD	RSD (%)	ReZ (Ω)	SD	RSD (%)	-ImZ (Ω)	SD	RSD (%)	-Zphase (^o^)	SD	RSD (%)
**IL-6**												
**TNT**/Ab	5567	52	0.9	1100	53	4.8	5457	93	1.7	27	1.4	5.1
5	5459	86	1.6	960	68	7.1	5374	84	1.6	28	1.3	4.6
10	5618	83	1.5	954	74	7.8	5536	74	1.3	30	1.9	6.3
25	6684	64	1.0	1256	46	3.7	6667	65	1.0	32	1.7	5.3
50	6830	76	1.1	1333	36	2.7	6699	87	1.3	33	1.4	4.3
100	7047	75	1.1	1156	83	7.2	6952	84	1.2	36	1.8	5.0
250	7743	92	1.2	1593	109	6.8	7577	62	0.8	39	1.5	3.9
500	8203	108	1.3	1372	89	6.5	8087	85	1.1	39	1.4	3.6
1000	8490	120	1.4	1567	77	4.9	8345	58	0.7	42	1.9	4.5
2500	9372	184	2.0	1917	114	5.9	9174	137	1.5	44	1.4	3.2
**IL-8**												
**TNT**/Ab	4507	43	1.0	822	57	6.9	4432	73	1.6	81	1.5	1.8
5	5831	69	1.2	1045	74	7.1	6159	58	0.9	79	1.9	2.4
10	6268	71	1.1	1018	56	5.5	6286	83	1.3	79	1.9	2.4
25	7051	98	1.4	1273	68	5.3	7253	59	0.8	78	1.7	2.2
50	7946	70	0.9	1535	82	5.3	7694	77	1.0	71	1.5	2.1
100	8217	94	1.1	2734	95	3.5	7253	85	1.2	71	1.7	2.4
150	8884	141	1.6	2881	85	2.9	8403	82	1.0	70	1.4	2.0
300	9669	109	1.1	3204	106	3.3	9123	95	1.0	69	1.9	2.7
600	11,839	118	1.0	5576	95	1.7	15,265	194	1.3	69	1.8	2.6
1200	16,252	168	1.0	9351	94	1.0	19,589	184	0.9	64	1.8	2.8
2500	21,706	182	0.8	14,594	134	0.9	21,499	192	0.9	56	1.8	3.2
**TNF** **α**												
**TNT**/Ab	4230	73	1.7	563	64	11.4	4193	65	1.6	82	1.8	2.2
5	4917	85	1.7	758	48	6.3	4802	59	1.2	79	2.1	2.7
10	6063	69	1.1	563	84	14.9	5724	128	2.2	80	1.9	2.4
25	7235	92	1.3	758	88	11.6	6842	95	1.4	80	1.8	2.2
50	7733	108	1.4	1185	92	7.8	7930	45	0.6	81	1.7	2.1
100	8253	125	1.5	1202	58	4.8	8157	184	2.3	81	2	2.5
250	9113	144	1.6	1256	93	7.4	7340	182	2.5	81	1.6	2.0
500	10,155	184	1.8	1883	183	9.7	9979	148	1.5	79	1.9	2.4
1000	12,081	199	1.6	2248	147	6.5	11,870	157	1.3	79	1.8	2.3
2500	18,082	176	1.0	2385	193	8.1	15,853	284	1.8	80	1.6	2.0
